# Eleven neurology-related proteins measured in serum are positively correlated to the severity of diabetic neuropathy

**DOI:** 10.1038/s41598-024-66471-6

**Published:** 2024-07-24

**Authors:** Emmanuel Bäckryd, Andreas Themistocleous, Anders Larsson, Torsten Gordh, Andrew S. C. Rice, Solomon Tesfaye, David L. Bennett, Björn Gerdle

**Affiliations:** 1 Pain and Rehabilitation Center, and Department of Health, Medicine and Caring Sciences, Linköping University, Linköping, Sweden; 2 Nuffield Department of Clinical Neurosciences, University of Oxford, Oxford, UK; 3 Department of Medical Sciences, Clinical Chemistry, Uppsala University, Uppsala, Sweden; 4 Department of Surgical Sciences, Uppsala University, Uppsala, Sweden; 5 Pain Research, Department of Surgery and Cancer, Faculty of Medicine, Imperial College London, London, UK; 6 Diabetes Research Unit, Sheffield Teaching Hospitals NHS Foundation Trust, Sheffield, UK

**Keywords:** Neuropathic pain, Neuroscience

## Abstract

About 20% of patients with diabetes suffer from chronic pain with neuropathic characteristics. We investigated the multivariate associations between 92 neurology-related proteins measured in serum from 190 patients with painful and painless diabetic neuropathy. Participants were recruited from the Pain in Neuropathy Study, an observational cross-sectional multicentre study in which participants underwent deep phenotyping. In the exploration cohort, two groups were defined by hierarchical cluster analyses of protein data. The proportion of painless vs painful neuropathy did not differ between the two groups, but one group had a significantly higher grade of neuropathy as measured by the Toronto Clinical Scoring System (TCSS). This finding was replicated in the replication cohort. Analyzing both groups together, we found that a group of 11 inter-correlated proteins (TNFRSF12A, SCARB2, N2DL-2, SKR3, EFNA4, LAYN, CLM-1, CD38, UNC5C, GFR-alpha-1, and JAM-B) were positively associated with TCSS values. Notably, EFNA4 and UNC5C are known to be part of axon guidance pathways. To conclude, although cluster analysis of 92 neurology-related proteins did not distinguish painful from painless diabetic neuropathy, we identified 11 proteins which positively correlated to neuropathy severity and warrant further investigation as potential biomarkers.

## Introduction

Neuropathic pain is defined as pain arising from a lesion or disease of the somatosensory nervous system^1^. Diabetes is one of the diseases which can cause neuropathic pain. About 20% of diabetic patients suffer from chronic pain with neuropathic characteristics^2^. Such neuropathic pain is associated with the development of distal symmetrical polyneuropathy (DSP)^3–5^. However, 50–75% of patients with diabetic DSP do *not* develop neuropathic pain^4,5^. Although there are well-known risk factors for the development of painful DSP (obesity, glycemic burden, and female sex^6,7^, and although there is an association between the severity of neuropathy and neuropathic pain^8^, it is still unclear why some patients with diabetic DSP develop neuropathic pain while others do not^9^. We have previously reported that, in patients with diabetic DSP, it was possible to identify a higher-inflammation subgroup in which high levels of hepatocyte growth factor, colony-stimulating factor 1, CD40 and 11 other inflammation-related proteins were associated with more severe neuropathy and higher pain intensity^10^.

Even when evidence-based first-line medicines such as tricyclics/duloxetine or gabapentinoids are prescribed adequately, only a minority of patients with neuropathic pain get substantial pain relief, the numbers needed to treat for > 30–50% pain relief ranging between 4 and 8^11^. For a majority of neuropathic pain patients, adequate pain relief will not be achieved. Importantly, there is a substantial “translational gap” between the achievements of preclinical (rodent) models of neuropathic pain and clinical reality^12–14^. In this context of deeply unmet medical needs, it has been suggested that human biomarker studies could be a way to bridge the translation gap^15^, e.g., by conducting proteomic studies^16^. Another way to explore the pathophysiology of different neurological conditions in humans is to use panels of pre-selected proteins such as, for instance, the Olink Neurology panel^17^. The 92 proteins of the Olink Neurology panel are a mix of established markers related to neurobiological processes and neurological diseases (e.g., neural development, axon guidance, synaptic function, or specific conditions such as Alzheimer’s disease), as well as some more exploratory proteins with broader roles in processes such as cellular regulation, immunology, development, and metabolism^18^.

The aim of this exploration and replication study was to investigate the multivariate associations between 92 neurology-related proteins measured in serum from diabetic DSP patients and clinical characteristics including pain intensity and neuropathy severity. Study participants were recruited as part of the Pain in Neuropathy Study (PiNS)^8^ and dichotomized into painful and painless diabetic DSP.

## Methods

### Overview and study rationale

Before any statistical analysis, the 190 study participants were randomized into an exploration (n = 95) and a replication (n = 95) cohort by using the random function in Microsoft Excel. The randomization was “frozen” in a pdf file on 20 May 2020. An underlying assumption in the present work is that (partly) different pathophysiological mechanisms might be at work in subgroups of patients suffering from painful diabetic DSP^10^. It has been suggested that many clinical disease entities may be umbrella terms encompassing several molecular mechanisms that share prominent signs and symptoms^19^. Therefore, our strategy in the present paper was to use cluster analysis to first define subgroups of patients based on the correlation structure of the analysed neurology-related proteins, the hypothesis being that such subgroups would be clinically meaningful. Such a strategy partly resembles that of Baron et al.^20^ who clustered peripheral neuropathic pain patients using quantitative sensory testing (QST). However, our subgrouping strategy is based not on psychophysical but on biological data, i.e., on protein levels. This approach is consistent with a systems medicine perspective, in which groups of interest are defined using “mechanism-based stratification”^19^ instead of the more conventional focus on signs and symptoms.

### Patients and clinical data

PiNS is an observational cross-sectional multicentre study in which participants underwent deep phenotyping that included neuropathy screening tools, extensive symptom and function questionnaires, neurological examination, nerve conduction studies, quantitative sensory testing, and skin biopsy for intraepidermal nerve fibre density assessment (IENFD) in a sub-set of patients^8^. Patients with diabetes mellitus aged above 18 years with diagnosed DSP, or patients with symptoms and signs suggestive of DSP were included. Exclusion criteria were pregnancy, coincident major psychiatric disorders, poor or no English language skills, severe pain at recruitment from a cause other than DSP (to prevent potential confounding influence on pain reporting as well as psychological and quality-of-life reported outcomes), patients with documented central nervous system lesions, or patients with insufficient mental capacity to provide informed consent or to complete questionnaires. Many of the study participants were recruited from primary care practices in London and Oxford. Study participants were also recruited from diabetes and other clinics at Chelsea and Westminster Hospital NHS Foundation Trust (London), Sheffield Teaching Hospitals and Oxford University Teaching Hospitals, neurology clinics at King’s College Hospital (London), and through advertisements.

Participants were included consecutively in PiNS until target number was reached. In PiNS, participants are dichotomized in painless and painful diabetic DSP. The methods and questionnaires have been previously described in detail^8^. Participants included in the present biomarker study were those where serum, and neuropathic pain grading according to IASP/NeuPSIG, were available^21^. We applied the NeupSIG grading system for neuropathic pain to pain in the feet as being the plausible anatomical distribution when separating those with painful versus painless diabetic neuropathy. In the present study, the following clinical variables were available for both painless and painful neuropathy patients:

- Age and sex
- Data pertaining to diabetes and metabolic control (Body Mass Index (BMI; kg/m^2^), diabetes type 1 or type 2, HbA1c)
- Data related to neuropathy—the Toronto Clinical Scoring System (TCSS) correlates with diabetic neuropathy severity^22^. Based on TCSS, patients can be classified as having no DSP (TCSS 0–5), mild DSP (TCSS 6–8), moderate DSP (TCSS 9–11), or severe DSP (TCSS 12–19)^23^.
- Douleur Neuropathique en 4 Questions (DN4) which can be used as a screening tool for neuropathic pain^24^.

Clinical variables available in patients with painful neuropathy were Brief Pain Inventory (BPI) severity scores^25^, Neuropathic Pain Symptoms Inventory (NPSI)^26^, and PainDetect^27^.

### Protein data

A 10 ml blood sample (BD Vacutainer SST Tubes) was drawn from each participant. After 30 min, to allow blood to clot, the sample was centrifuged at 3000 rpm for 10 min at a temperature of 4 °C. Serum was then aliquoted into 1.8 ml Nunc CryoTubes and stored at – 80 °C.

The Olink Neurology panel (product number 95801, v. 8012) provides a high-throughput, multiplex immunoassay enabling the analysis of 92 neurology-related protein biomarkers at the same time using a Proximity Extension Assay (PEA) technology^28^^,^^29^^,^^30^^,^^31^. PEA means that a pair of oligonucleotide-labelled antibodies bind to their respective target protein. When the two antibodies are close to each other, a polymerase chain reaction (PCR) is initiated which is then quantified by real time PCR. Results are expressed as Normalized Protein eXpression (NPX), which is *relative* quantification between samples, on a Log2 scale. A high NPX value equals a high protein concentration. Because NPX is a Log2 scale, a difference of 1 in NPX means a doubling of protein concentration. If needed, NPX values can be converted into a linear scale according to 2^NPX^ = linear NPX. A complete list of the 92 neurology-related proteins, including their UniProt ID, is found in Supplemental Digital Content 1 (see Supplementary Information 1). The URL leading to validation data provided by Olink is available here: https://www.olink.com/content/uploads/2021/09/olink-neurology-validation-data-v2.1.pdf (Access date April 30, 2024).

### Statistics

Data are expressed as median (IQR), unless stated otherwise. SIMCA (version 16, Sartorius Stedim Biotech, Umeå, Sweden) was used for multivariate data analysis (MVDA). SPSS (version 26, IBM Corporation, Route 100 Somers, New York, USA) was used for all other analyses (Mann–Whitney U test, Chi-Square test, Spearman’s rho for bivariate correlations, and multiple linear regression (MLR), as appropriate). A significance level of 0.05 was chosen.

The same procedures were conducted in the exploration (n = 95) and in the replication (n = 95) cohorts. Details concerning MVDA methodology^32,33^ have been described in previous publications^30,34–39^. Briefly, we performed principal component analysis (PCA), hierarchical clustering analysis (HCA) and, based on the groups defined by HCA, orthogonal partial least squares (-discriminant analysis) (OPLS and OPLS-DA). PCA is a technique that models the correlation structure of a dataset, and thereby enables the identification of multivariate outliers^32,33^. Principal components (PC) extract relevant information found in the data, reducing a high-dimensional space (high number of variables) to a few “summary variables”. After outlier detection with PCA (strong outliers defined as Hotelling’s T2>>T2Crit(99%) and moderate outliers as DModX>2*DCrit), we applied a bottom-up HCA to the principal component score vectors using the default Ward linkage criterion to identify relevant subgroups of patients. HCA complements PCA in the sense that while PCA identifies distinct clusters in multivariate space, HCA can find subtle clusters. In the resulting dendrogram, interesting patient subgroups were identified, and clinical data were compared between subgroups to ascertain the clinical relevance of the subgroups. Then, OPLS-DA was performed using group belonging as Y-variables and protein data as predictors (X-variables). To identify the proteins most relevant for group discrimination, the OPLS-DA models analyzed and identified associations between the X-variables and group belonging. X-variables with |p(corr)|≥ 0.5 are usually considered important for group discrimination^32^, the sign of p(corr) denoting the direction of the association (described in text in each case). However, in some cases, a tougher cut-off of |p(corr)|≥ 0.6 was used instead in this study. P(corr) is the loading of each X-variable scaled as a correlation coefficient that is comparable between models. MVDA analyzes all variables simultaneously, using the overall correlation pattern present in the data, hence separating information from “noise”. Hence, the protein data in the present study were *not* primarily analyzed by multiple univariate testing, thereby minimizing the multiple testing problem. In a third step, both cohorts were analyzed together in an OPLS model with TCSS as outcome (Y) variable—see text below for rationale. For this third step, a false discovery rate (FDR) at the 10% level was applied using the Benjamini–Hochberg procedure^40^.

### Ethics

The study was approved by the National Research Ethics Service of the United Kingdom (No.:10/H07056/35). All study participants signed written consent before participating. The research was conducted in accordance with the Declaration of Helsinki.

## Results

Two proteins out of 92 had > 20% missing values and were therefore excluded from all analyses. All results are based on the remaining 90 proteins. An overview of clinical data in painless vs. painful patients is presented in Table 1.

**Table 1:** Clinical data in painless vs. painful diabetic neuropathy in 190 patients.

	Painless neuropathy n = 96	Painful neuropathy n = 94	p-value
Age (years)	72 (67–78)	71 (61–76)	0.042*
Sex (% females)	30%	30%	0.950
BMI (kg/m^2^)	27.7 (25.1–31.4)	30.7 (27.2–35.2)	< 0.001*
HbA1c %	7.1 (6.5–7.8)	7.9 (7.0–8.8)	0.002*
Type 2 diabetes %	92%	91%	0.965
TCSS	8 (6–11)	13 (10–16)	< 0.001*
DN4	2 (1–3)	6 (4–7)	< 0.001*
BPI pain average	N.A.	6 (4–7)	N.A.
BPI worst	N.A.	8 (6–8)	N.A.
BPI least	N.A.	3 (2–5)	N.A.
BPI now	N.A.	5 (3–7)	N.A.
BPI severity subscore	N.A.	5.3 (4–6.5)	N.A.
NPSI total	N.A.	7 (4.2–11.6)	N.A.
PainDetect	N.A.	18 (12.5–22)	N.A.

Data are expressed as median (25th–75th percentiles) except for sex.

BMI, Body Mass Index; TCSS, Toronto Clinical Scoring System; DN4, Douleur Neuropathique en 4 Questions; BPI, Brief Pain Inventory 0–10; NPSI total, Neuropathic Pain Symptoms Inventory, total value.

*Denotes statistical significance at the 0.05 level.

### First phase of protein analyses: exploration cohort

The exploration cohort consisted of 95 patients. Two patients (ID 30483 and ID 30519) were excluded because of quality warning from Olink Bioscience (Uppsala, Sweden). On the remaining 93 patients, a PCA was done using the 90 proteins as X-variables (4 PCs, R^2^ = 0.52, Q^2^ = 0.38); no outlier was found. By HCA, 2 groups were defined (Group 1, n = 37 and Group 2, n = 56). Then, the clinical variables were compared between the two groups, i.e., we wanted to see if they seemed clinically meaningful. In the exploration cohort (Table 2, left side), TCSS was significantly higher in Group 2 (11 (8–15) vs. 8.5 (6–12), p = 0.027). The other clinical variables did not differ between groups in the exploration cohort. Finally, an OPLS-DA was done with group belonging (Group 1 vs 2) as Y-variable; the model had 2 latent variables (one predictive and one orthogonal component), R^2^ = 0.69, Q^2^ = 0.57, and p < 0.001 by CV-ANOVA. The proteins most responsible for group discrimination, i.e., with |p(corr)|≥ 0.6 for the first (predictive) latent variable, are listed in Table 3^**,**^ left column.

**Table 2:** Clinical data in the exploration and replication cohorts (left and right, respectively), in each case comparing Groups 1 and 2 as defined by hierarchical cluster analysis in each cohort.

	Exploration cohort			Replication cohort		
	Group 1	Group 2	P-value	Group 1	Group 2	p-value
Age (years)	69 (61–76)	71 (64–78)	0.179	70(61–73)	74(68–78)	0.003*
Sex (% females)	33%	30%	0.764	29%	30%	0.911
BMI (kg/m^2^)	28.1 (24.3–33.3)	29.4 (26.6–35.3)	0.164	29.1 (27.0–32.4)	29.0 (26.5–32.3)	0.637
HbA1c%	7.4 (7.0–8.8)	7.5 (6.6–8.4)	0.343	7.9 (6.6–8.7)	7.3 (6.6–8.1)	0.550
Painful neuropathy (%)	44%	50%	0.603	45%	52%	0.510
TCSS	8.5 (6–12)	11 (8–15)	0.027*	10 (8.5–12)	12.5(9–14)	0.016*
DN4	3 (1–5.5)	4(2–5)	0.291	4(1–5.5)	4(2.5–6)	P = 0.292

Data are expressed as median (25th–75th percentiles) unless specified otherwise.

BMI, body mass index; TCSS, Toronto Clinical Scoring System; DN4, Douleur Neuropathique en 4 Questions.

*Denotes statistical significance at the 0.05 level.

**Table 3:**
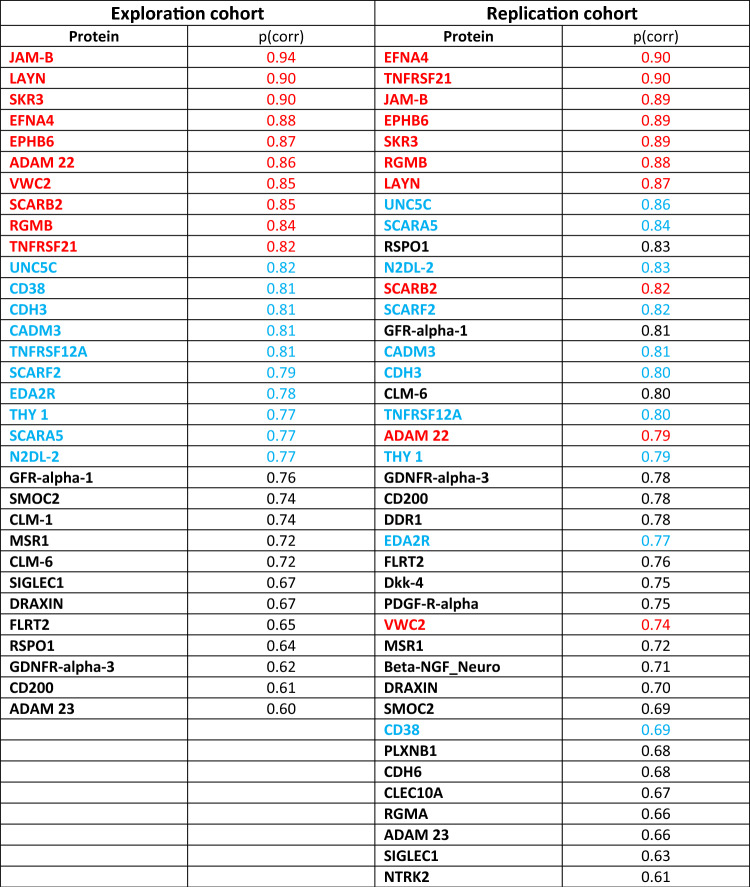
Proteins responsible for group discrimination (Group 1 vs 2) in the exploration cohort (left columns) and the replication cohort (right columns) in falling order of p(corr).

To illustrate the overlap of results, the top 10 proteins of the exploration cohort are in red in both columns, and the top 11–20 proteins of the exploration cohort are in blue in both columns. The p(corr) values in the table all have a positive sign, indicating higher levels in Group 2 compared to Group 1. All top 20 proteins in the exploration cohort had a p(corr) > 0.68 in the replication cohort.

### Second phase of protein analyses: replication cohort

After an initial PCA on the 95 patients of the replication cohort using the 90 proteins as X-variables, one patient (ID 30087) was excluded for being a multivariate outlier by Hotelling’s T2. Hence, the new PCA model had 94 patients, with 4 PCs, R^2^ = 0.53, Q^2^ = 0.40. By HCA, 2 groups were defined (Group 1, n = 31 and Group 2, n = 63). Then, we tested the hypothesis that TCSS scores would differ between Group 1 and 2. We found that, just as in the exploration cohort, Group 2 had significantly higher TCSS scores: (12.5 (9–14) vs. 10 (8.5–12), p = 0.016) (Table 2, right side). Age was also significant (p = 0.003) (Table 2). Hence, the findings concerning TCSS were replicated. Finally, an OPLS-DA was done with group belonging (Group 1 vs 2) as Y-variable; the model had 1 latent variable (i.e., one predictive component), R^2^ = 0.56, Q^2^ = 0.53, and p < 0.001 by CV-ANOVA. The proteins most responsible for group discrimination, i.e., with |p(corr)|≥ 0.6, are listed in Table 3^,^ right side. Notably, 70% of the top ten proteins of the exploration cohort were top 10 in the replication cohort (see text in red in Table 3) and 85% of the top 20 proteins of the exploration cohort were top 20 in the replication cohort (see text in blue in Table 3).

### Third phase: in-depth analysis of TCSS in all patients

In third phase of the study, we focused on TCSS, using both cohorts taken together (n = 186; TCSS was missing in one patient). Using TCSS as Y-variable and the 90 proteins as X-variables, we computed an OPLS model which had 2 latent variables (one predictive and one orthogonal), R^2^ = 0.31, Q^2^ = 0.10, p < 0.001 by CV-ANOVA. The proteins most associated with TCSS are tabulated in Table 4; the overlap with the two OPLS-DA models in Table 3 is also illustrated.

**Table 4:** Top 22 proteins* associated with Toronto Clinical Scoring System (TCSS) in OPLS model.

Proteins associated with TCSS (n = 186)	p(corr) with TCSS as Y-variable	Is this protein also part of the top 22 proteins of the exploration cohort?	Is this protein also part of the top 22 proteins of the replication cohort?
EDA2R	0.64	Yes ☑	No
TNFRSF12A	0.62	Yes ☑	Yes ☑
SCARB2	0.62	Yes ☑	Yes ☑
N2DL-2	0.59	Yes ☑	Yes ☑
SKR3	0.59	Yes ☑	Yes ☑
EFNA4	0.58	Yes ☑	Yes ☑
LAYN	0.56	Yes ☑	Yes ☑
CLM-1	0.55	No	No
CD38	0.54	Yes ☑	No
UNC5C	0.52	Yes ☑	Yes ☑
GFR-alpha-1	0.51	Yes ☑	Yes ☑
JAM-B	0.50	Yes ☑	Yes ☑
CLM-6	0.48	No	Yes ☑
Beta-NGF	0.46	No	No
CDH3	0.46	Yes ☑	Yes ☑
SMOC2	0.44	Yes ☑	No
SCARA5	0.42	Yes ☑	Yes ☑
MSR1	0.42	No	No
TNFRSF21	0.42	Yes ☑	Yes ☑
VWC2	0.42	Yes ☑	No
ADAM 22	0.42	Yes ☑	Yes ☑
THY 1	0.40	Yes ☑	Yes ☑

p(corr) values are all positive, corresponding to positive correlations with TCSS. Included in the table is also an illustration of the overlap with the top 22 proteins of the two previous OPLS-DA models according to Table 3 (exploration and replication), see the two columns furthest to the right.

*Note: To enable a comprehensive comparison with the exploration and replication cohorts, 0.4 was here chosen as cut-off value for |p(corr)|, rendering a list of 22 proteins listed in falling order of p(corr). However, consistently with the pre-defined cut-off value of 0.5 (see “Methods”), the main findings of the study are the 11 proteins with |p(corr)|of at least 0.5, excluding EDA2R, see text.

Because of a possible age issue (see Table 2), we next computed a new OPLS model, with age as Y variable to be able to exclude age-related proteins from the results of Table 4. This age model had 3 latent variables (one predictive and two orthogonal), n = 186, R^2^ = 0.64, Q^2^ = 0.45, p < 0.001 by CV-ANOVA. We found 4 proteins with |p(corr)|≥ 0.4 (see note in Table 4 for choice of p(corr) level), indicating a possible association with age, and EDA2R, the top protein in Table 4^**,**^ had the highest p(corr) in the age-model (positive correlation with age and p(corr) = 0.5). Therefore, EDA2R was excluded from the main results of the study (see below).

Hence, the 11 proteins with p(corr) ≥ 0.5 in Table 4 (i.e., excluding EDA2R as per above) were the main findings of the present study, and all correlated positively with TCSS in multivariate space. Additionally, we also computed bivariate correlations between TCSS and the 11 proteins, finding that the 11 proteins had highly significant correlations with TCSS, with rho ranging from 0.19 to 0.32. These 11 correlations remained significant when applying a false discovery rate (FDR) of 10% (calculated for 90 bivariate correlations between TCSS and each protein). The 11 proteins also all inter-correlated significantly with each other (all p-values < 0.001) with rho ranging from 0.51 to 0.88—hence confirming the validity of this cluster of proteins which together correlated positively with TCSS (as per the OPLS model above). The 11 proteins are described briefly together with their Uniprot ID in Table 5. To further descriptively get a sense the effect sizes involved, we calculated the percentage increases of median values of linearized NPX in those having severe DSP compared to those *not* having DSP (defined as TCSS < 6, n = 18), see Table 5. Rho-values (i.e., correlation with TCSS) are also listed in Table 5. Also, the relationship between the 11 proteins and TCSS is visualized in Supplemental Digital Content 2 (see Supplementary Figures).

**Table 5:** Cluster of proteins correlating positively with Toronto Clinical Scoring System (TCSS).

	Protein name	Uniprot ID	Rho *** (p-value)	Increase* (%)	Function**
TNFRSF12A	Tumor necrosis factor receptor superfamily member 12A	Q9NP84	0.29 (p < 0.001)	31	Weak inducer of apoptosis in some cell types, promotes angiogenesis and the proliferation of endothelial cells. TNFRSF12A may also modulate cellular adhesion to matrix proteins, as well as play some role in the positive regulation of axon extension
SCARB2	Lysosome membrane protein 2	Q14108	0.26 (p < 0.001)	37	Animal studies suggest that this protein may participate in membrane transportation and the reorganization of the endosomal/lysosomal compartment. In humans, CD36 appears to be involved in host-virus interactions and may have a role in hand, foot, and mouth disease linked to enterovirus-71 infections
N2DL-2	NKG2D ligand 2	Q9BZM5	0.25 (p < 0.001)	37	It functions as a stress-induced ligand for NKG2D receptor and activates natural killer (NK) cells by inducing multiple signalling pathways. N2Dl-2 is expressed in fetal tissues and a number of cancer cell lines but is not typically expressed in normal adult tissues
SKR3	Serine/threonine-protein kinase receptor R3	P37023	0.29 (p < 0.001)	30	A type I cell-surface receptor for the TGF-beta superfamily of ligands. It is an important regulator of normal blood vessel development, and mutations in the gene for this protein are associated with hemorrhagic telangiectasia type 2, also known as Rendu-Osler-Weber syndrome 2
EFNA4	Ephrin-A4, or EPH-related receptor tyrosine kinase ligand 4	P52798	0.25 (p < 0.001)	22	Ephrin proteins have been implicated in mediating developmental events, especially in the nervous system and in erythropoiesis and are thought to be crucial for cellular migration, repulsion and adhesion during these processes. Ephrin-A4 has a suggested role in axon guidance, the chemotactic process by which migration of an axon growth cone is directed to a specific target site
LAYN	Layilin	Q6UX15	0.25 (p < 0.001)	33	Layilin may have a role in cell adhesion and motility. There may also be indications of some connection to neurofibromatosis 2, an inherited condition characterised by multiple forms of benign intracranial tumors
CLM-1	CMRF35-like molecule 1	Q8TDQ1	0.32 (p < 0.001)	42	Mediates negative regulatory signals by recruiting SHP1 and inhibits osteoclast formation. There may be some indications of associations between CLM-1 and post-traumatic epilepsy
CD38	ADP-ribosyl cyclase/cyclic ADP-ribose hydrolase 1	P28907	0.28 (p < 0.001)	34	The protein is expressed at high levels in some tumors, such as malignant lymphoma and neuroblastoma. Loss of CD38 function is associated with a number of issues, including impaired immune responses, metabolic disturbances, and behavioral modifications including social amnesia
UNC5C	Netrin receptor UNC5C, or Protein unc-5 homolog C	O95185	0.19 (p = 0.008)	22	Netrins are secreted proteins that direct axon extension and cell migration during neural development. Following netrin binding, UNC5C mediates axon repulsion of neuronal growth cones in the developing nervous system. It also has an independent function in corticospinal tract axon guidance
GFR-alpha-1	GDNF family receptor alpha-1, or RET ligand 1 and TGF-beta-related neurotrophic factor receptor	P56159	0.29 (p < 0.001)	32	Is the functional receptor for glial cell-derived neurotropic factor (GDNF), and in this capacity plays a key role in the control of neuron survival and differentiation. GFR-alpha-1 may be involved in development of the enteric nervous system of the gastrointestinal tract
JAM-B	Junctional adhesion molecule B, or Junctional adhesion molecule 2	P57087	0.22 (p = 0.003)	22	It acts as an adhesive ligand for interacting with a variety of immune cell types and may play a role in lymphocyte homing to secondary lymphoid organs. It is thought to promote lymphocyte transendothelial migration and may also be involved with endothelial cell polarity, by associating to cell polarity protein PAR-3, together with JAM3

Notes: *The percentages in the “increase” column indicate descriptively how much larger the median levels (in linearized normalized protein expression, NPX) were in the severe DSP group (n = 76) compared to no DSP (n = 18). For details about NPX, see “Statistics” section. **This short description is based on information on the Olink website, www.olink.com, accessed 23 May 2020. ***Correlation coefficient with TCSS.

Using the 11 proteins as per above, we computed a PCA model, n = 186, 1 PC, R^2^ = 0.77, Q^2^ = 0.72. We used the scores of the PC of the PCA model as a “summary” variable of the 11 proteins, here called PC1_11prot. We did a multiple linear regression (MLR) with TCSS as outcome variable (dependent variable) and with the following variables as predictors: PC1_11prot, sex, age, BMI, and HbA1c. The MLR model was significant (adjusted R^2^ = 0.155 and p < 0.001) and PC1_11prot was significant (p = 0.001 with a positive coefficient, i.e., a positive correlation between PC1_11prot and TCSS) when adjusted for sex, age, BMI and HbA1c.

### Fourth phase: in-depth analysis of BPI scores in patients with painful neuropathy

Finally, we did an in-depth analysis of BPI scores in patients with *painful* neuropathy. In the exploratory cohort, only “BPI now” differed when comparing Groups 1 and 2, Group 2 (n = 28) having statistically significant higher levels than Group 1 (n = 15): 6,5 (4–8) vs. 3 (1, 5–6), p = 0.019; this remained significant at FDR 10%, the critical value being 0.02. In the replication cohort however, none of the BPI scores differed between Groups 1 and 2. “BPI now” in the replication cohort was 6 (4–7) in Group 1 (n = 14) vs 4 (2–6) in Group 2 (n = 33), p = 0.205 (i.e., a non-significant tendency for “BPI now” to be *lower* in Group 2). Hence, the findings of “BPI now” in the exploratory cohort could not be replicated.

## Discussion

A panel of 92 neurology-related proteins was used to investigate potential biomarkers of painful and painless diabetic DSP in a deeply phenotyped cohort. We found that 11 proteins were associated with the severity of neuropathy (but not with the presence of neuropathic pain). These 11 proteins have a variety of biological functions such as inflammatory processes, growth factors, adhesion molecules and axon guidance (Table 5). Neuropathic pain is known to positively correlate with the severity of peripheral neuropathy^8^. However, given its complex aetiology involving multiple pathophysiological drivers in the central as well as peripheral nervous system^41^, it is not surprising that we may find molecular correlates of neuropathy severity that are independent of neuropathic pain.

One biological process that was highlighted in our findings was axon guidance with the identification of EFNA4 and UNC5C. Ephrins, to which EFNA4 belongs, is one of five known families of axon guidance proteins^42^. Axon guidance pathways seem to be involved in diabetic DSP^43^. Interestingly, Evdokimov et al.^44^ studied EFNA4 in *skin* biopsies from fibromyalgia patients vs. controls, finding that the expression of EFNA4 was higher in fibromyalgia patients. Axon guidance proteins are detected by a structure at the tip of growing axons—the growth cone^42^. Different receptors are present on the growth cone, one of them being UNC5C, which is another of the main findings listed in Table 5. UNC5C in turn binds to the netrin family of axon guidance proteins^42^. In diabetic DSP, both nerve degeneration and regeneration are present^45^, and the question therefore arises if EFNA4 and UNC5C can perhaps be seen as potential biomarkers for nerve de- and/or re-generation in this setting? This is of course highly speculative and should be investigated in further studies. It should also be noted that ephrins and netrins have been implicated in central processes related to (neuropathic) pain^46–49^, and that UNC5C has been investigated in the context of endometriosis-related (and supposedly neuropathic) pain^50^. Moreover, although it was not associated to TCSS as per Table 4, the ephrin EPHB6 was nonetheless a main finding in both the exploration and the replication cohorts as per Table 3.

Given previous finding about chronic inflammation in painful and painless diabetic DSP^10^, our findings about GFR-alpha-1 and CD38 are also interesting (Table 5). CD38 is immunomodulatory^51^ and has been deemed to be a possible pharmacological target. In mice, Gil and co-workers studied CD38 in the context of osteoarthritis, concluding that inhibition of CD38 could potentially be a novel therapeutic approach for the treatment of osteoarthritis and associated pain^52^. GFR-alpha-1 is the receptor for GDNF—which in turn is connected to inflammation^53^. GDNF has also been shown to have neuroprotective actions on sensory neurons following traumatic axotomy^54^ and in experimental models of diabetic neuropathy^55^. Hence, although the pain literature contains scarce information about the potential biomarkers listed in Table 5, at least five of them can be related to neuropathy/pain in different ways. It should be noted that Neurofilament light chain, a potential biomarker ford diabetic DSP^56^, was not part of the panel of proteins in the present study.

In our opinion, the present study has some obvious strengths. Before any statistical analysis of the data, the material was dichotomized into two cohorts, enabling us to implement an exploration-replication strategy which confirmed that the subgroup characterized by high levels of the proteins listed in Table 3 had higher levels of neuropathy as expressed by TCSS (Table 2). Hence, although this paper is hypothesis-generating in the sense that it used a panel of neurology-related proteins (and thus no specific candidate proteins), there is also an element of confirmatory methodology inherent in the exploration-replication design. Moreover, as in previous work^10^, we used an unbiased clustering approach to subgroup the patient on biological grounds. Hence, instead of merely comparing painful and painless participants, we stratified the material according to a systems biology perspective—and this stratification was then shown to be clinically relevant, albeit not directly pain-wise. The idea behind this approach is that there might be different mechanisms at play in different patients who have the same symptoms and signs, and that a simple comparison based on phenotype might thus blur the picture more than a comparison of clusters based on biology. Whether this “mechanism-based stratification”^19^ approach is really advantageous will of course have to be confirmed or falsified in future studies. Concerning the fact that we did not find a *pain* signal, it is of course important to remember the subjective and biopsychosocial nature of the pain experience. Elucidating the biology underlying the subjective experience is a task as difficult as it is important^15,57,58^. Pain biomarker studies are undertaken with methods from different fields, e.g., imaging methods^59^, electrophysiology^60^, or ‘omics^16^. Recent advances concerning the role of calcitonin gene-related peptide (CGRP) in migraine^61^ should give pain researchers some confidence that the search for biomarkers reflecting the pathophysiology of different chronic pain conditions is hopefully not a futile task.

Obvious study limitations include the cross-sectional design and the possibility of confounders such as for instance concomitant medication (although our findings seem robust when it comes to the influence of sex, age, BMI and HbA1c). The possibility of there being a systematic error in the material cannot be ruled out. Also, even if our results would turn out to be valid in the sense that they really reflect neuropathy-related pathophysiology, it is still important to consider whether the described “fingerprint” relates directly to the pathophysiology of neuropathy, or if perhaps it is a risk factor that was present prior to the development of neuropathy. A third possibility could be that the fingerprint is an epiphenomenon, perhaps more related to co-morbidities such as insomnia or depression. Disentangling the contribution of potentially mutually interacting factors is a challenge and will require longitudinal studies. Fourth, when measuring multiple analytes in a single experiment, antibody specificity is an important issue to be aware of. The PEA technology^28^^,^^29^^,^^30^^,^^31^ builds on dual recognition, i.e., a *pair* of oligonucleotide-labelled antibodies have to bind to their respective target protein to generate a signal, leading to higher specificity compared to methods based on a single antibody. This fact notwithstanding, the question marks raised by the specificity issue remain a major limitation in the present work. The findings should therefore be interpreted cautiously, warranting further replication studies using alternative methods of detection.

To conclude, in Table 5 we present a list of 11 inter-correlated proteins who were positively correlated to the severity of neuropathy in DSP patients but not to the presence of neuropathic pain. These may have potential as novel biomarkers for diabetic neuropathy which are increasingly important as new understanding of axon degeneration has led to novel drug targets to prevent axon degeneration. The validity and clinical relevance of these putative neuropathy biomarkers will need to be confirmed in future longitudinal studies.

### Supplementary Information

Supplementary Information 1.

Supplementary Figures.

## Data Availability

Data cannot be made publicly available because of the lack of ethical permission. If the corresponding author is contacted, reasonable data requests can be considered.
