# Supplementary material for: Eleven neurology-related proteins measured in serum are positively correlated to the severity of diabetic neuropathy
Source: Sci Rep. 2024 Jul 24;14:17068. doi: 10.1038/s41598-024-66471-6 (PMC11269577; doi:10.1038/s41598-024-66471-6)
Supplement: Supplementary file 1 — Supplementary Information 1. [file 41598_2024_66471_MOESM1_ESM.pdf]

Olink®

Protein assay list

Olink® Target 96 Neurology

Product number: 95801

ADP-ribosyl cyclase/cyclic ADP-ribose hydrolase 1 (CD38)	P28907	Ephrin type-B receptor 6 (EPHB6)	O15197
Alpha-2-macroglobulin receptor-associated protein (Alpha-2-MRAP)	P30533	Ephrin-A4 (EFNA4)	P52798
		Epithelial discoidin domain-containing receptor 1 (DDR1)	Q08345
BDNF/NT-3 growth factors receptor (NTRK2)	Q16620	Ezrin (EZR)	P15311
Beta-nerve growth factor (Beta-NGF)	P01138	Fc receptor-like protein 2 (FcRL2)	Q96LA5
Bone morphogenetic protein 4 (BMP-4)	P12644		
Brevican core protein (BCAN)	Q96GW7	Galectin-8 (gal-8)	O00214
Brorin (VWC2)	Q2TAL6	GDNF family receptor alpha-1 (GFR-alpha-1)	P56159
Cadherin-3 (CDH3)	P22223	GDNF family receptor alpha-3 (GDNFR-alpha-3)	O60609
Cadherin-6 (CDH6)	P55285	Glial cell line-derived neurotrophic factor (GDNF)	P39905
		Glypican-5 (GCP5)	P78333
Carboxypeptidase A2 (CPA2)	P48052	Granulocyte Colony-Stimulating Factor (G-CSF)	P09919
Carboxypeptidase M (CPM)	P14384	Granulocyte-macrophage colony-stimulating factor receptor subunit alpha (GM-CSF-R-alpha)	P15509
Cathepsin S (CTSS)	P25774		
Cell adhesion molecule 3 (CADM3)	Q8N126	Granzyme A (GZMA)	P12544
Cell surface glycoprotein CD200 receptor 1 (CD200R1)	Q8TD46	Growth/differentiation factor 8 (GDF-8)	O14793
		Hydroxyacylglutathione hydrolase, mitochondrial (HAGH)	Q16775
CMRF35-like molecule 1 (CLM-1)	Q8TDQ1	Interleukin-5 receptor subunit alpha (IL-5R-alpha)	Q01344
CMRF35-like molecule 6 (CLM-6)	Q08708	Interleukin-12 (IL-12)	P29460, P29459
Contactin-5 (CNTN5)	O94779		
C-type lectin domain family 1 member B (CLEC1B)	Q9P126	Junctional adhesion molecule B (JAM-B)	P57087
C-type lectin domain family 10 member A (CLEC10A)	Q8IUN9	Kynureninase (KYNU)	Q16719
Cytotoxic and regulatory T-cell molecule (CRTAM)	O95727	Latexin (LXN)	Q9BS40
Dickkopf-related protein 4 (Dkk-4)	Q9UBT3	Layilin (LAYN)	Q6UX15
Dipeptidyl peptidase 1 (CTSC)	P53634	Leucine-rich repeat transmembrane protein FLRT2 (FLRT2)	O43155
Disintegrin and metalloproteinase domain-containing protein 22 (ADAM 22)	Q9P0K1	Leukocyte-associated immunoglobulin-like receptor 2 (LAIR-2)	Q6ISS4
Disintegrin and metalloproteinase domain-containing protein 23 (ADAM 23)	O75077	Linker for activation of T-cells family member 1 (LAT)	O43561
Draxin (DRAXIN)	Q8NBI3	Lysosome membrane protein 2 (SCARB2)	Q14108

Table continues on reverse ▶

Macrophage scavenger receptor types I and II (MSR1)	P21757
MAM domain-containing glycosylphosphatidylinositol anchor protein 1 (MDGA1)	Q8NFP4
Matrilin-3 (MATN3)	O15232
Mesencephalic astrocyte-derived neurotrophic factor (MANF)	P55145
Microtubule-associated protein tau (MAPT)	P10636
N-acylethanolamine-hydrolyzing acid amidase (NAAA)	Q02083
Neprilysin (NEP)	P08473
Netrin receptor UNC5C (UNC5C)	O95185
Neuroblastoma suppressor of tumorigenicity 1 (NBL1)	P41271
Neurocan core protein (NCAN)	O14594
Neuronal cell adhesion molecule (Nr-CAM)	Q92823
Neuropilin-2 (NRP2)	O60462
Neutral ceramidase (N-CDase)	Q9NR71
Nicotinamide/nicotinic acid mononucleotide adenylyltransferase 1 (NMNAT1)	Q9HAN9
NKG2D ligand 2 (N2DL-2)	Q9BZM5
NT-3 growth factor receptor (NTRK3)	Q16288
OX-2 membrane glycoprotein (CD200)	P41217
Platelet-derived growth factor receptor alpha (PDGF-R-alpha)	P16234
Plexin-B1 (PLXNB1)	O43157
Plexin-B3 (PLXNB3)	Q9ULL4
Poliovirus receptor (PVR)	P15151
Protogenin (PRTG)	Q2VWP7
Repulsive guidance molecule A (RGMA)	Q96B86
RGM domain family member B (RGMB)	Q6NW40
Roundabout homolog 2 (ROBO2)	Q9HCK4
R-spondin-1 (RSPO1)	Q2MKA7
Scavenger receptor class A member 5 (SCARA5)	Q6ZMJ2
Scavenger receptor class F member 2 (SCARF2)	Q96GP6
Secreted frizzled-related protein 3 (sFRP-3)	Q92765
Serine/threonine-protein kinase receptor R3 (SKR3)	P37023
Sialic acid-binding Ig-like lectin 9 (Siglec-9)	Q9Y336
Sialoadhesin (SIGLEC1)	Q9BZZ2
SPARC-related modular calcium-binding protein 2 (SMOC2)	Q9H3U7
Sphingomyelin phosphodiesterase (SMPD1)	P17405
Tenascin-R (TN-R)	Q92752
Testican-1 (SPOCK1)	Q08629
Thy-1 membrane glycoprotein (THY 1)	P04216
Transmembrane protease serine 5 (TMPRSS5)	Q9H3S3
Tumor necrosis factor receptor superfamily member 12A (TNFRSF12A)	Q9NP84
Tumor necrosis factor receptor superfamily member 21 (TNFRSF21)	O75509
Tumor necrosis factor receptor superfamily member 27 (EDA2R)	Q9HAV5
WAP, Kazal, immunoglobulin, Kunitz and NTR domain-containing protein 1 (WFIKKN1)	Q96NZ8

For more details visit www.olink.com/neurology

www.olink.com

For research use only. Not for use in diagnostic procedures.
This product includes a license for non-commercial use. Commercial users may require additional licenses. Please contact Olink Proteomics AB for details.
There are no warranties, expressed or implied, which extend beyond this description. Olink Proteomics AB is not liable for property damage, personal injury, or economic loss caused by this product.
Olink® is a registered trademark of Olink Proteomics AB.

© 2017–2022 Olink Proteomics AB. All third party trademarks are the property of their respective owners.
Olink Proteomics, Dag Hammarskjölds väg 52B , SE-752 37 Uppsala, Sweden
1035, v2.0, 2022-06-16
