# Supplementary material for: Eleven neurology-related proteins measured in serum are positively correlated to the severity of diabetic neuropathy
Source: Sci Rep. 2024 Jul 24;14:17068. doi: 10.1038/s41598-024-66471-6 (PMC11269577; doi:10.1038/s41598-024-66471-6)

Supplemental Digital Content 2

**SUPPLEMENTARY FIGURE S1, panel a-K**: Scatter plots between Toronto Clinical Scoring System (TCSS) and each of the 11 proteins that are the main findings of the study (expressed as NPX, see paper for explanation).

A)


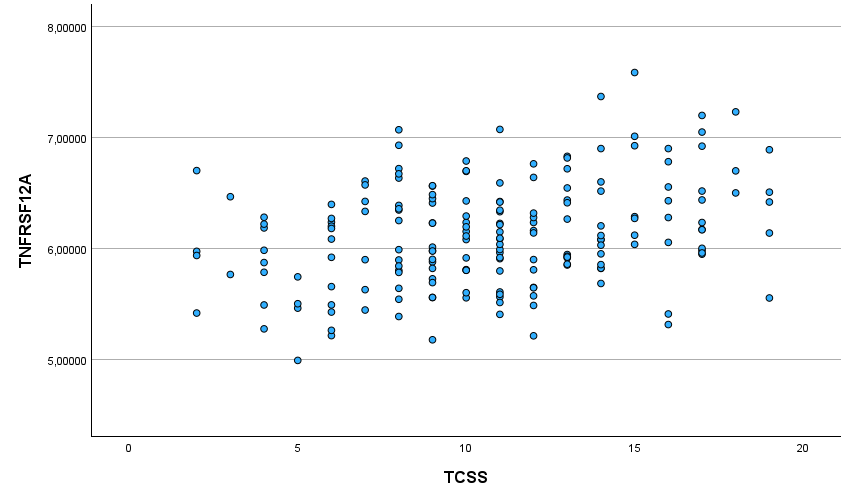


B)


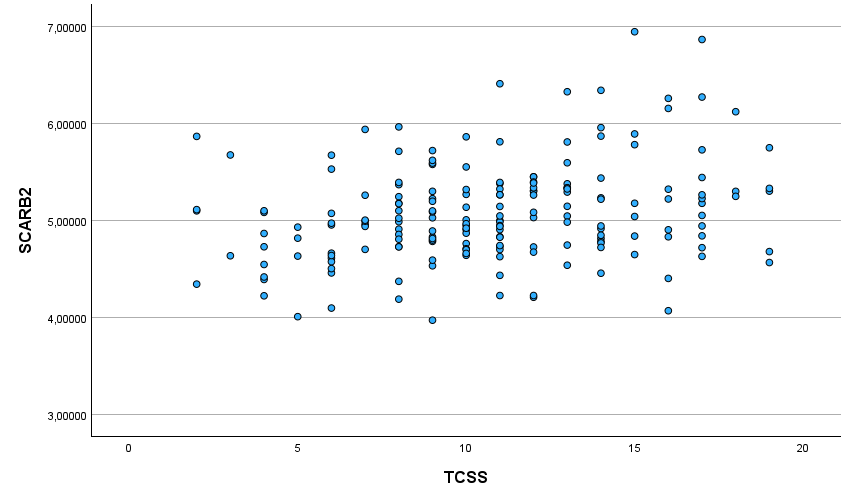


C)


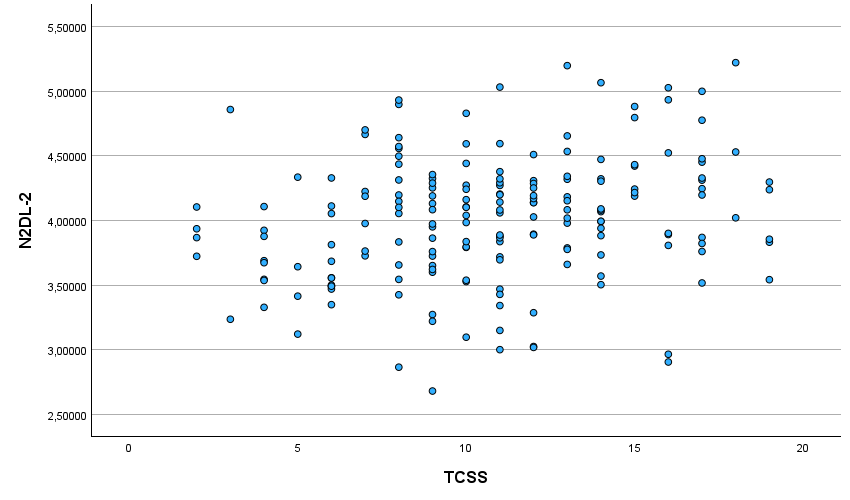


D)


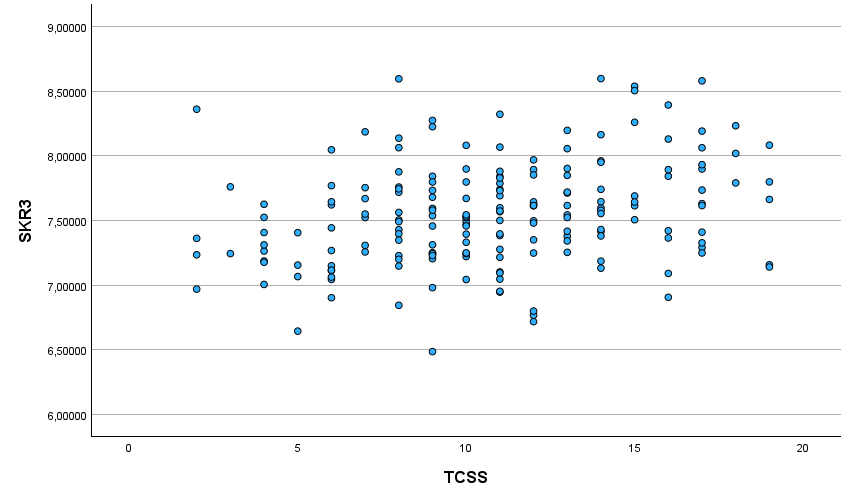


E)


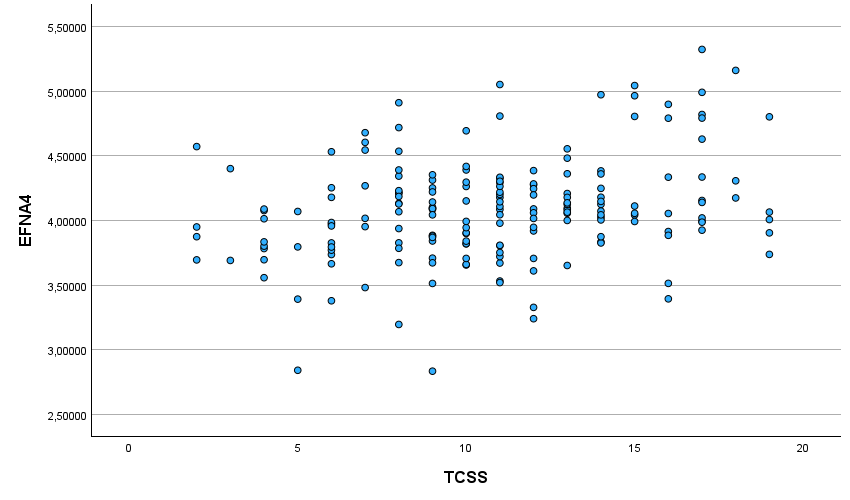


F)


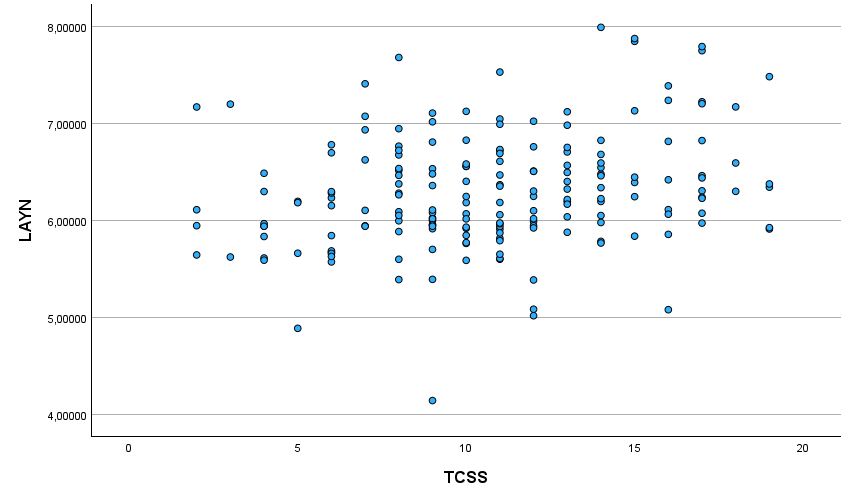


G)


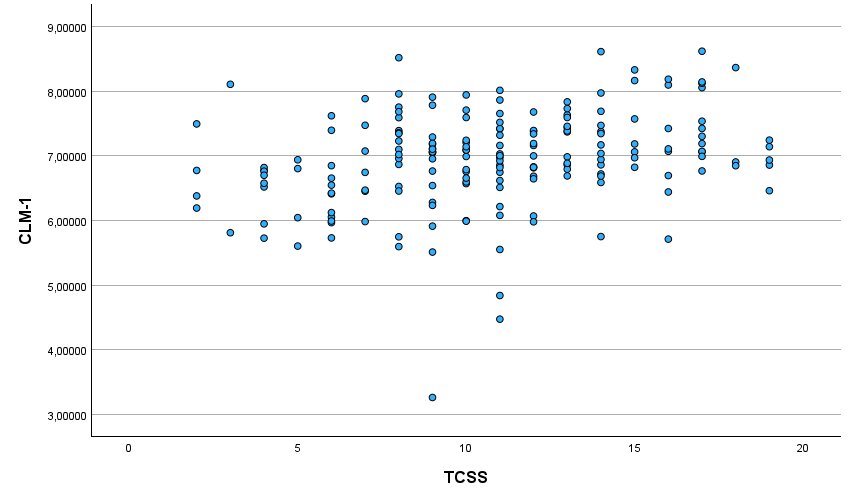


H)


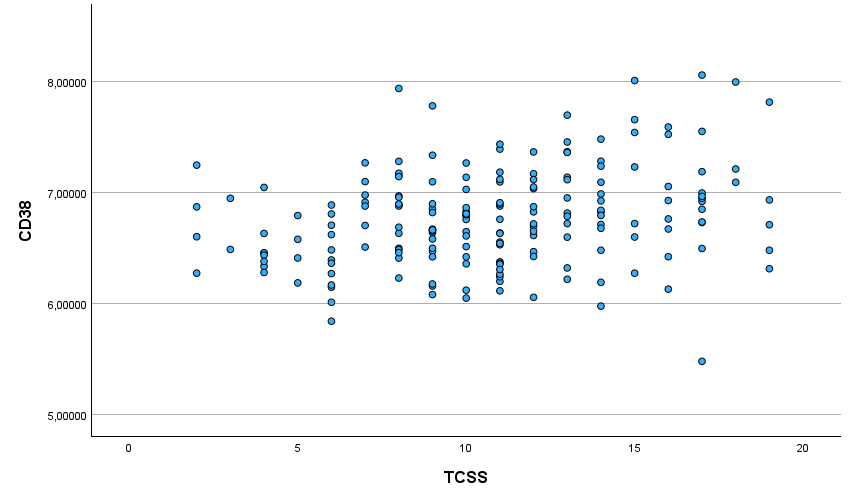


I)


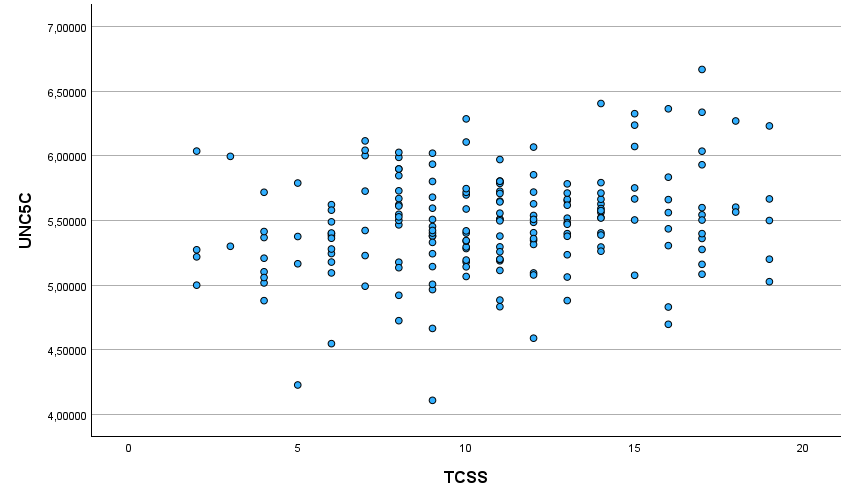


J)


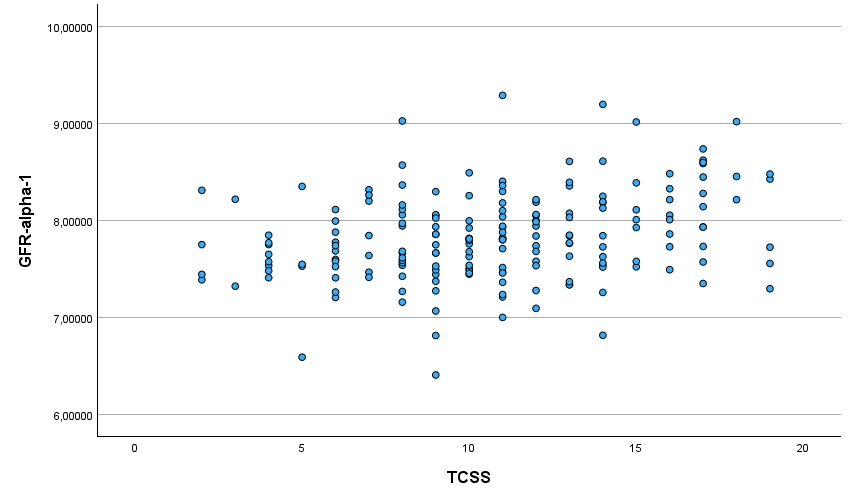


K)


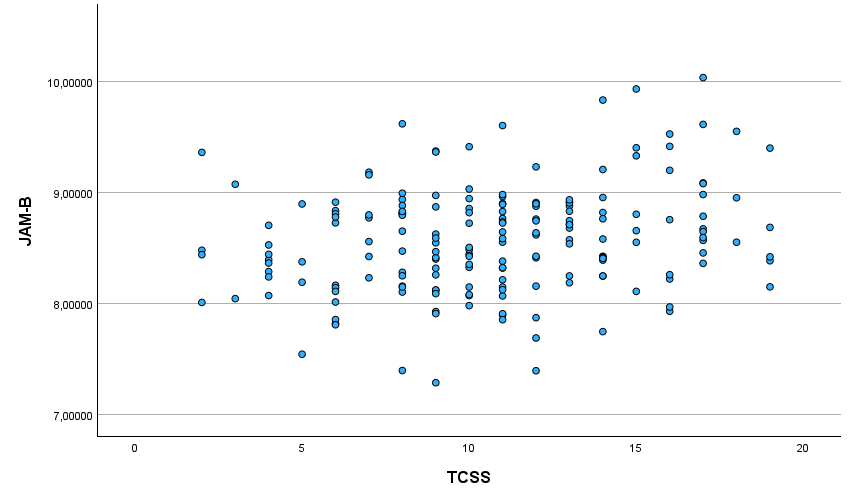


**SUPPLEMENTARY FIGURE S2, panel a-K:** Boxplots showing levels of each of the 11 proteins (expressed as NPX, see paper for explanation) in patients with no, mild, moderate or severe neuropathy according to Toronto Clinical Scoring System (TCSS).

Notes:

- DPN=Diabetic Polyneuropathy
- No DPN (n=19), i.e., TCSS 0-5
- Mild DPN (n=36), i.e, TCSS 6-8
- Moderate DPN (n=56), i.e., TCSS 9-11
- Severe DPN (n=75), i.e., TCSS 12-19

A)


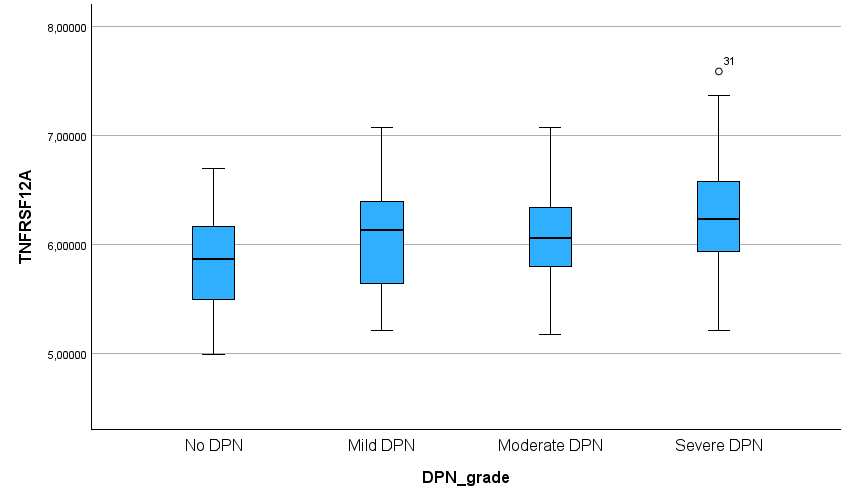


B)


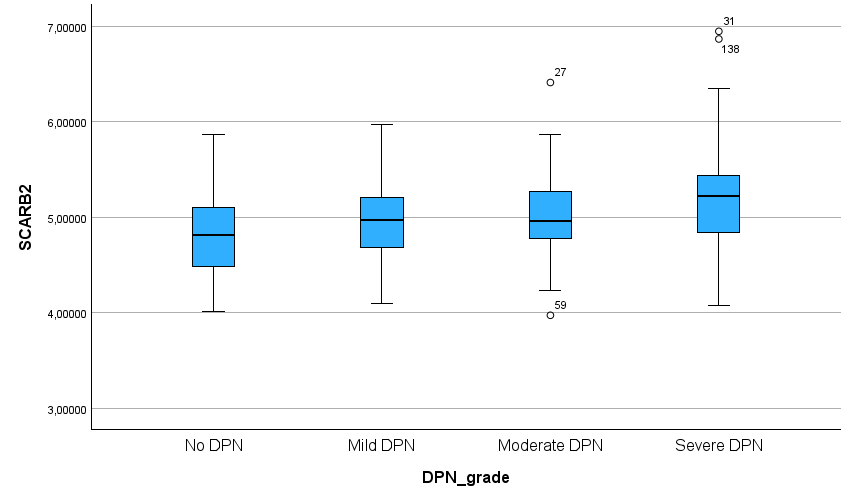


C)


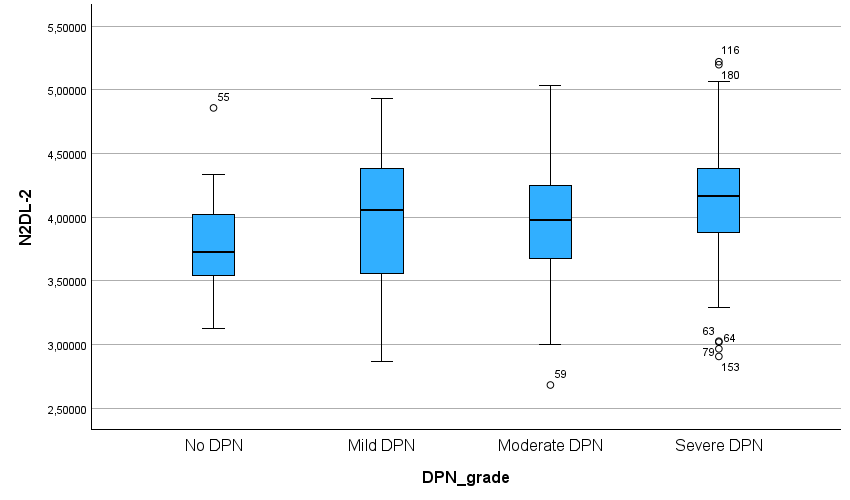


D)


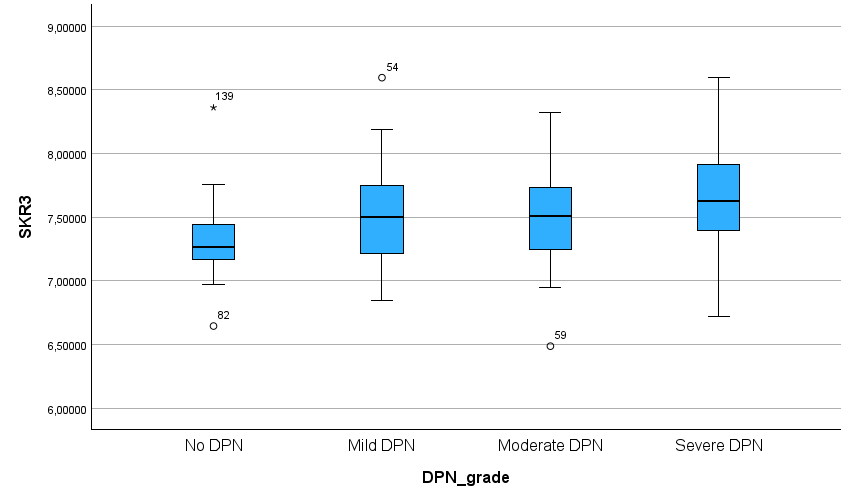


E)


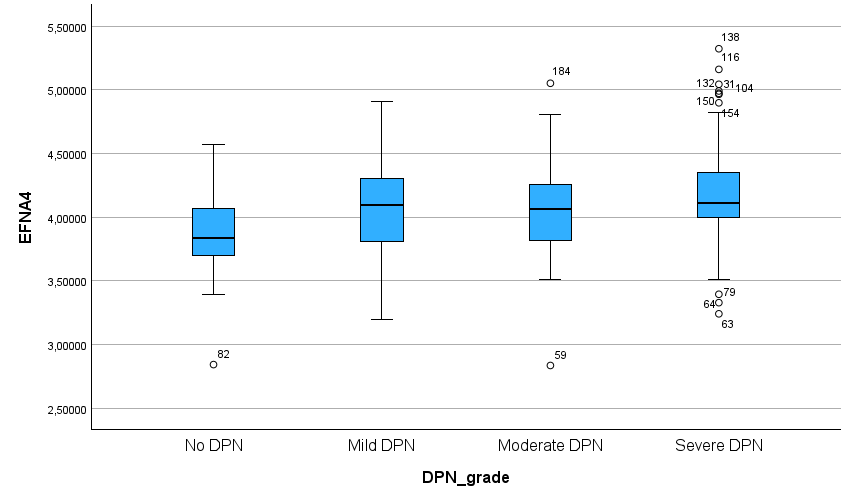


F)


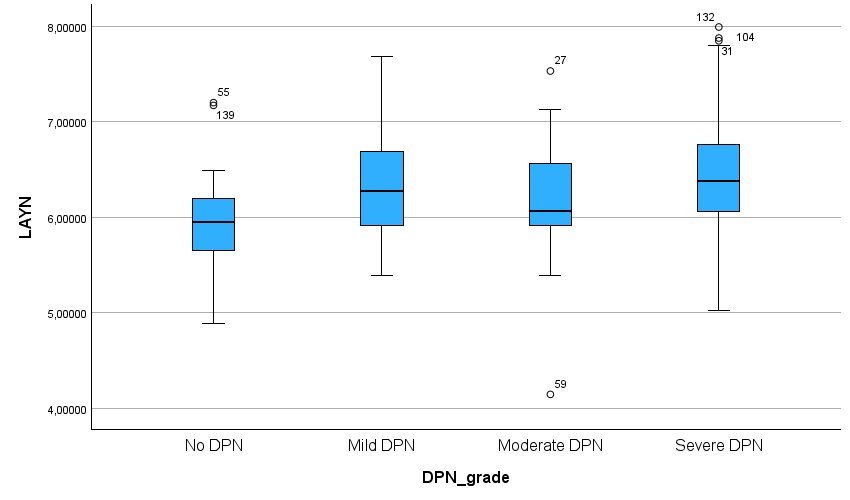


G)


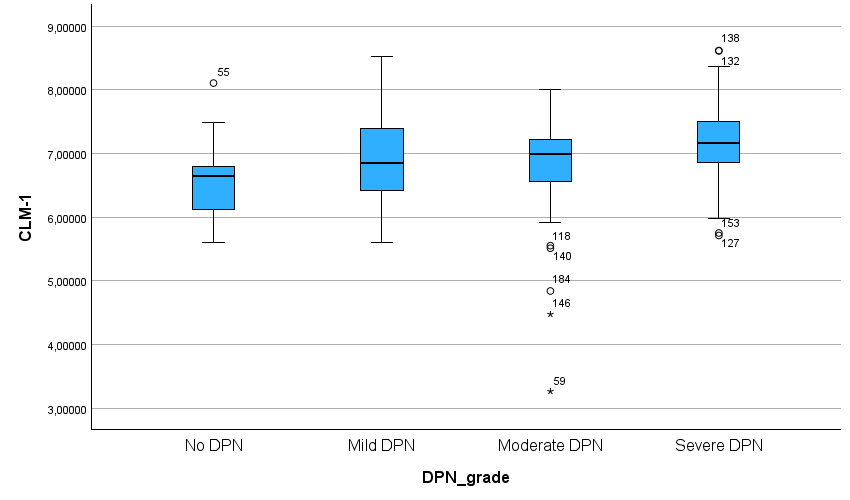


H)


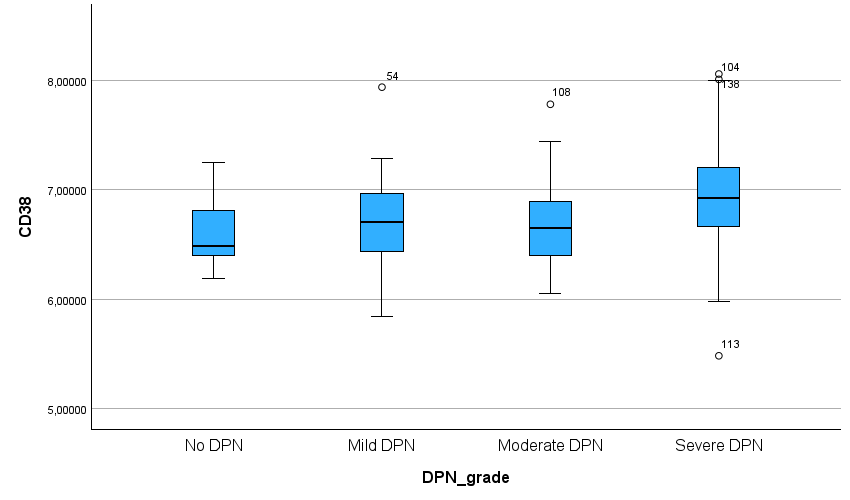


I)


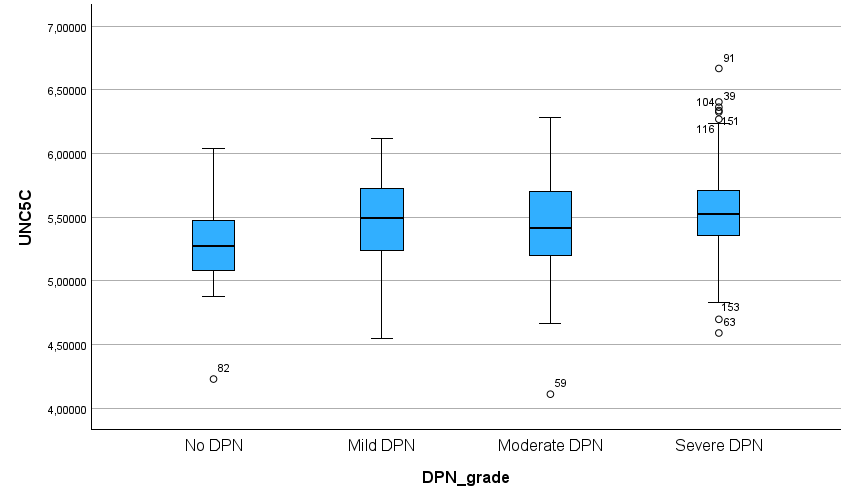


J)


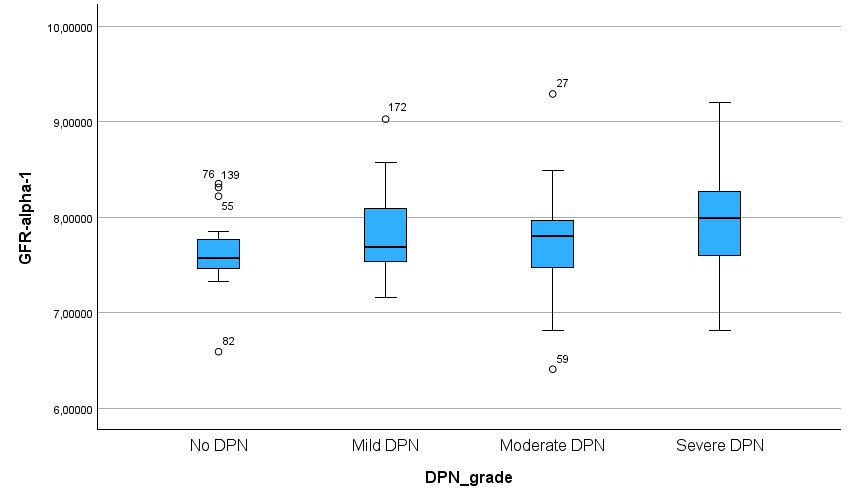


K)


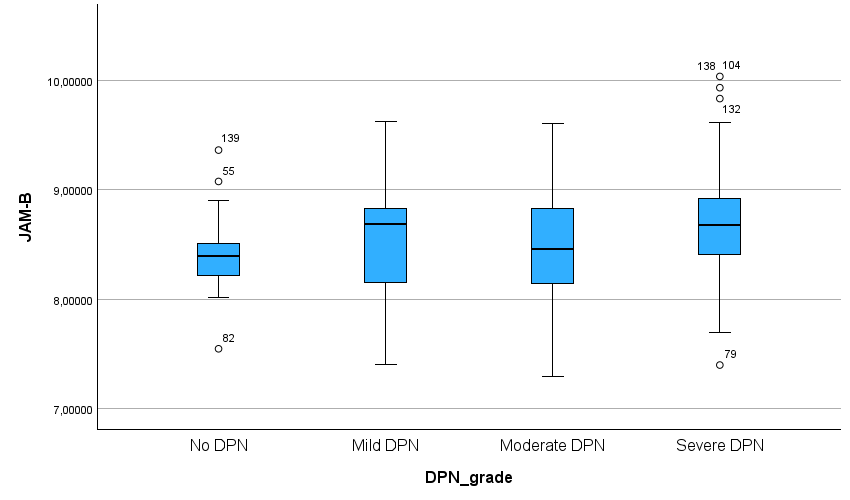

Supplement: Supplementary file 2 — Supplementary Figures. [file 41598_2024_66471_MOESM2_ESM.docx]
